# A Baby Born with Ectopia Cordis, Omphalocele, Cleft Lips and Palate: A Case Report

**DOI:** 10.31729/jnma.7153

**Published:** 2022-02-28

**Authors:** Nischal Shrestha

**Affiliations:** 1 Department of Paediatrics, Nobel Medical College and Teaching Hospital, Biratnagar, Nepal

**Keywords:** *cleft lip*, *cleft palate*, *ectopia cordis*, *omphalocele*

## Abstract

Ectopia cordis is a rare congenital defect with the prevalence of 5 to 8 per million live births. Here we report a rare case of preterm female live birth with ectopia cordis associated with omphalocele, cleft lip, and palate. In this case, 14+ weeks ultrasound did not show any fetal abnormalities and parents were unaware of the condition until 35+ weeks when ultrasound detected the anomaly a few days before delivery. After delivery, they didn't give consent for further intervention which led to neonatal mortality 3 hours after birth. If the condition was diagnosed in time, an earlier intervention could have been done.

## INTRODUCTION

Ectopia Cordis (EC) is a rare congenital malformation that occurs due to a defect in the fusion of the anterior chest wall resulting in complete (the naked heart is displaced outside thoracic cavity without pericardium) or partial displacement of the heart (heart can be seen pulsating through the skin) outside the thoracic cavity.^1^ The management of such case is surgery which is challenging because of very few cases of long term survivors. Here we present a case report of EC with omphalocele, cleft lip and palate.

## CASE REPORT

A 18-year-old, unbooked primigravida, O positive blood group, Mongolian race presented with complaints of decreased fetal movement for three days. The mother was not suffering from any illness during pregnancy. There was no history of infection, intake of any teratogenic drugs, or exposure to any radiation in the antenatal period. There was no family history of congenital anomalies. There was no history of consanguinity. Her 14+ weeks of gestation (WOG) ultrasonography (USG) showed no gross fetal abnormality. But her USG at 35+^2^ WOG showed fetal abnormalities. Both the woman and her family were aware of the USG report.

**Figure 1:**
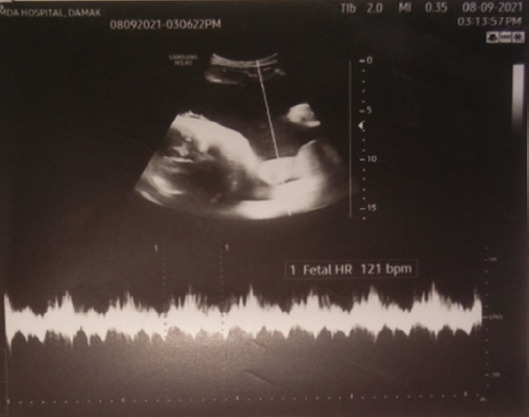
Ectopia cordis.

**Figure 2:**
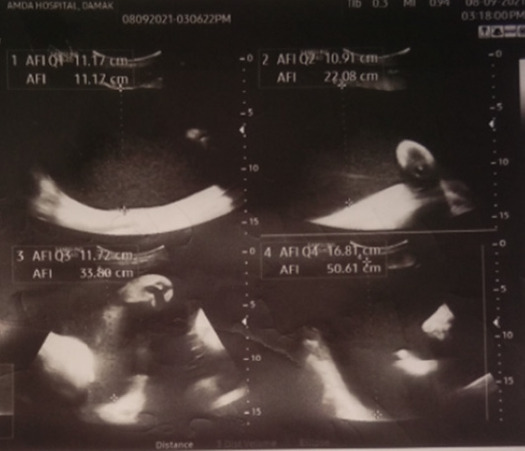
Polyhydramnios.

The USG findings can be summarized as: in the 14+ WOG, no gross fetal abnormality was evident. The presentation was breech with anterior placentation and adequate liquor volume: in the 35^th^ week, the heart of the fetus appeared outside the thoracic cavity (abdomino-thoracic type of Ectopia Cordis) (Figure 1), solid structure mainly liver appeared away from the anterior abdominal wall (Omphalocele), upper lip region showed lucency (cleft palate), amniotic fluid index (AFI) was 50.8cm (polyhydramnios) with breech presentation (Figure 2).

**Figure 3:**
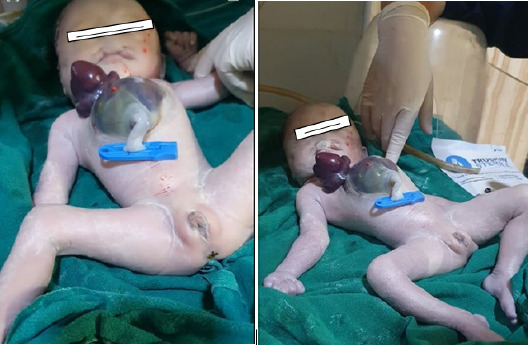
Ectopia cordis with omphalocele and cleft lip.

A live preterm female baby of 2kgs was born at the 35+^5^ WOG by spontaneous vaginal delivery at a tertiary care centre of Nepal. The newborn had an APGAR score of seven and eight in the 1^st^ and 5^th^ minutes, respectively. An anterior thoracoabdominal defect with extrathoracic heart, a cleft sternum, omphalocele, bilateral cleft lip, and palate was recognized at birth. On gross examination, the heart was totally outside of the thoracic cavity without pericardium protection. There was an abdominal wall defect with supraumbilical omphalocele where the umbilical cord was attached at the lower part of the defect (Figure 3).

Other abnormal features included telecanthus (broad nasal bridge). The mother and her family refused for admission of the baby. As a result of progressive respiratory dysfunction, neonatal death occurred in the first three hours of life. The family did not allow to perform an autopsy examination. The neonate died in her first three hours before any surgical intervention was done.

## DISCUSSION

Haller, et al. in 1706 described the term ectopia cordis for the first time.^2^ But, the cause is yet unknown; however, it has been reported that EC may be associated with chromosomal disorders including trisomy 18, Turner syndrome, 46XX, and 17q+, viral infections and exposure to teratogens.^2,3^ On physical examination, in this case, telecanthus was noted which might be associated with chromosomal disorders like Turner's syndrome, Down's syndrome,^4^ but the chromosomal analysis could not be done as consent was not given.

EC occurs mostly sporadically; also, in this case, no family history was known. Only a few cases of familial EC have been reported.^5^ The reported prevalence is five to eight per million live births. It constitutes 0.1% of all congenital heart diseases. It has shown its association with cardiac anomalies such as ventricular septal defects (VSDs), atrial septal defects (ASDs), pulmonary stenosis (PS), right ventricular (RV) diverticulum, double outlet right ventricle (DORV), and tetralogy of Fallot.^6^ Very few cases of EC with double outlet right ventricle have been reported till now.^7,8^ In this case, we couldn't find out intracardiac anomaly as we could not perform echocardiography. Non-cardiac disorders such as omphalocele, gastroschisis, scoliosis, cleft palate/lip, and central nervous system disorders have also been seen with EC.^9^ Similar to reports by Taksande and Morales, this case had omphalocele and cleft lip/palate associated with EC.^9,10^

Embryogenesis reveals that the development of the ventral body wall begins with differentiation and proliferation of mesoderm followed by its lateral migration by the eighth day of the embryonic life. The heart initially develops in cephalic location but reaches its ultimate position by lateral folding and ventral flexing of the embryo at about the 16^th^-17^th^ day. By the 9^th^-week midline fusion occurs and the formation of the thoracic and abdominal cavities completes. If there is a complete failure of midline fusion at this stage, then complete ventral evisceration occurs. Isolated EC occurs if there is incomplete midline fusion.^6,11^ EC can be classified on the basis of the location of the heart into 5 types: cervical (5%), cervicothoracic and thoracic (65%), thoracoabdominal (20%), and abdominal (10%).^12^ If along with thoracoabdominal EC, there occurs lower sternal defect, anterior diaphragmatic hernia, midline supraumbilical abdominal wall defect, and intracardiac defects, then the condition is termed as Pentalogy of Cantrell.^13^ In this case, we could see thoracoabdominal EC which usually accompanies Cantrell's syndrome but we could not find out if there was an intracardiac defect as we were unable to do an echocardiogram. A similar case with the Pentalogy of Cantrell was reported by Kumar B, et al.^14^

Prenatally we can diagnose EC using 2D ultrasound as early as in 10-12 weeks of pregnancy.^15^ For the firsttrimester diagnosis, 2D is sufficient, while 3D has greater usefulness in the 2^nd^ and 3^rd^ trimesters.^16,17^ Omphalocele can be detected early at the 12^th^ week of menstrual age. But in this case, the 14+ weeks scan did not show any gross abnormality. Abnormality was identified only in 35^+^ weeks USG. MRI in conjunction with prenatal echocardiography allows optimal assessment of the fetus with ectopia cordis and omphalocele.^18^ We can diagnose cleft lip and palate using 2D and 3D USG in the 2^nd^ and 3^rd^ trimesters. Data of accurate diagnosis in 1st trimester is lacking.^19^

The complete EC (as our case) where the heart is completely outside the thoracic cavity, is a neonatal emergency. The initial management of EC is concerned with immediate covering the heart and exposed abdominal contents (omphalocele). For this, silastic prosthesis is recommended. Before closing the abdominal wall, we should evaluate and correct intracardiac defects.^20^ Surgical repair of omphalocele is done within the first 72 hours of life. Commonly surgical repair of cleft lip and palate is done at 12^th^ month of life. The management of such cases is a multistep, complicated, and expensive process, with a poor prognosis. So, even if the case was detected earlier and intervention would have been taken in time, the probability of a better outcome would be low. In this case, the family came to know about the fetal condition through ultrasound just a few days before delivery at our hospital and after the delivery, they refused the admission of the baby to NICU. Therefore, no intervention could be tried and the baby died within 3 hours due to respiratory failure.

As the patient did not follow up from the beginning, detailed information regarding the case could not be reported in this case report. Also, we could not diagnose the case early and intervention could not be done. Therefore, WHO guidelines for a pregnant woman regarding antenatal care visit and ultrasonography is recommended. If any serious abnormality is detected, termination can be a choice so that it will not be a burden for the mother and also the baby after birth will not have to suffer.
